# Painting Nature on the Wing

**DOI:** 10.3201/eid1201.AC1201

**Published:** 2006-01

**Authors:** Polyxeni Potter

**Affiliations:** * Centers for Disease Control and Prevention, Atlanta, Georgia, USA

**Keywords:** Winslow Homer, James Edward Kelly, John LaFarge, landscape, Lloyd Goodrich

**Figure:**
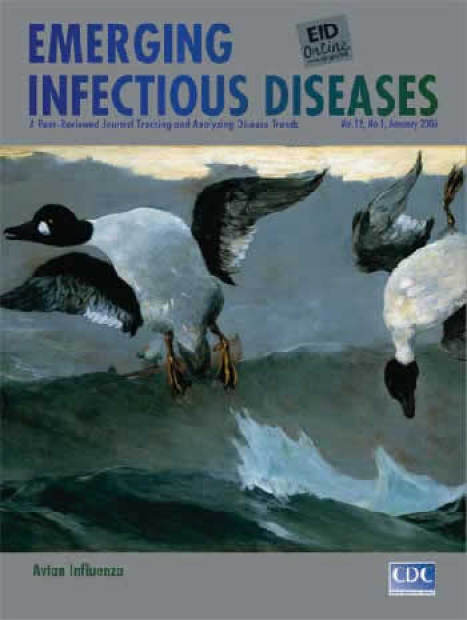
Winslow Homer (1836–1910). Right and Left (1909). Oil on canvas (0.718 m × 1.229 m). National Gallery of Art, Washington, DC. Gift of the Avalon Foundation

"The Sun will not rise, or set, without my notice, and thanks," wrote Winslow Homer to his brother Charles, putting into words what drove his art (*1*). All his life, the artist "noticed"—not just the rising and setting sun from his home near the ocean at Prouts Neck, Maine, but everything around him: people; scenery of his hunting and fishing trips; wildlife, which he painted with passion; objects and details others would never imagine the subject of art; and the effect, movement, and inner life of these. As he gave drawing instructions to his friend and fellow artist James Edward Kelly (1855–1933), Homer once said, "You should practice drawing old shoes and getting their character….You should practice drawing high hats…. There is a great deal of drawing in a high hat, to get not only its curves, but its delicate variations in the outline which gives it style" (*2*).

In Cambridge, Massachusetts, where he grew up, Homer was exposed to art and encouraged to draw at an early age. His mother, Henrietta Benson, a successful illustrator of flowers and other still life, taught him watercolor painting and foresaw his "future greatness." Hers were the only works, besides his own, found in Homer's studio after his death. As a youth, he was apprenticed to a Boston lithographer and later took evening classes at the National Academy of Design in New York, but he was primarily self-taught, his career marked by continued growth (*3*).

At age 21, Homer became a freelance illustrator and in no time he was working for the prestigious Harper's Weekly. Soon, the Civil War broke out. Sent to the battlefields as artist-correspondent, he turned his observant eye not to war action and combat but to soldiers' plight. He sketched from life at camp then converted sketches into engravings at his studio in New York. Documenting drab labor behind the lines and the soldiers' loneliness and alienation gained him national recognition.

"Quite late this man went to Europe and studied there and found things ready to his hand, but I do not know what more he got beyond what he had already," wrote John LaFarge (1835–1910), Homer's friend and fellow artist (*2*). Homer's time in Paris, after the war, and in Cullercoats, an English fishing village and artists' colony on the North Sea, had little influence on his style. "A great man," LaFarge believed, "…has left for us what I think is the only record of absolutely American Yankee expression" (*2*).

"You will see, in the future I will live by my watercolors," Homer remarked when, already accomplished in the weightier world of oils, he returned to the more direct medium of his childhood (*4*). The freshness and lightness of watercolor lent itself more naturally to his vision. He painted common people and bucolic scenes, the innocence of an era quickly disappearing from 19th-century America. "Barbarously simple," said Henry James. "He has chosen the least pictorial features of the least pictorial range of scenery and civilization as if they were every inch as good as Capri or Tangier; and, to reward his audacity, he has incontestably succeeded" (*4*).

Homer's interest in watercolor painting matured at his Prouts Neck studio, a refurbished stable off the family estate. In addition to "wearing out the deck" of his home pacing as he observed ocean storms, he traveled to Nassau, Cuba, Florida, Bermuda, interpreting the bright Caribbean light on the exotic scenery, omitting human figures, showing them in their struggle against the forces of nature, or displaying their fragility against vast expanses of sea and sky. He became one of the best watercolorists in the world.

An avid sportsman and naturalist, Homer was also master of the wildlife genre, best at both observing and identifying with the sporting landscape. He traveled with palette and brushes, painting nature on the wing. From Prouts Neck to the Adirondacks, from Quebec to the Florida Keys, he captured bird and trout, the woods, the whitetail, the common duck, identifying with the hunters and the hunted, marveling at and fearing nature, outlining a structured landscape that was uniquely simple and grand. "Homer," his biographer Lloyd Goodrich wrote, "…employed the affirmative elements of the American spirit…. He did for our painting what Walt Whitman did for our poetry—he made it native to our own earth and water" (*5*).

The illusion suggested by the artist's work is directed by him but mostly made by us," wrote LaFarge, suggesting that looking at a work of art is not passive for it involves the viewer's imagination (*2*). And since viewer cannot be separated from art, preconception or lack of "knowing" can interfere with appreciation. In his small circle of friends, Homer was dubbed "the obtuse bard." An allusion to his Hellenic namesake, this description fit a certain poetic license in his work to say one thing and mean another, as well as his often misunderstood attempts at humor, which, like his paintings, allowed multiple interpretations.

Right and Left, on this month's cover, was painted a year before Homer's death (*6*). The title is hunt jargon for using a double-barreled gun to shoot two ducks in rapid succession. The hunter, on the waves in some distance, is barely visible behind the flare of the shotgun. We witness the aquatic scene from the birds' perspective in the sky. Bird on the right, possibly struck first, falls limply toward the ocean. Bird on the left, in direct range, makes desperate attempt at exit as the second shot is fired. Or is the bird on the left stunned from being hit first, in the back, while the other bird is diving to escape?

The "in your face" travel of the birds and bullets adds dramatic immediacy. Agitated waters, a glaring eye, the rocking boat underline violence. Dislodged feather and ray of sunshine mark the fleeting moment. This scene, painted when death must have been on Homer's mind, seems the culmination of a lifetime of observation.

"Good metaphor," noted Aristotle in his Poetics, "implies an intuitive perception of the similarity of dissimilars," it implies likeness (*7*). A bird is not human, but a single element in its appearance can invoke humanity, just as a single element in a plant's appearance can distinguish its species. Homer's masterful hunting scene, commissioned as a sporting picture, is much more.

In a few brushstrokes, the artist delivers the ocean's power, the vastness of creation, conflict in the world, riddles in nature. He projects the birds and their plight, the hunter's unimportance, even as he fires the fatal shots. The ambiguity in their posture is the artist's ambivalence about which bird died first. The threat is imminent and inevitable. Death is certain.

The artist's vision holds yet more ambiguity today. The sporting ducks deliver as well as receive havoc. When they escape the double-barreled shotgun and fly off, they may carry with them nature's revenge, introducing new flu virus strains right and left: to domestic animals or directly to humans, increasing risks for new pandemics. As we stare into the hunter's barrel in Homer's painting, we could be the sitting ducks.
